# Associations between the development of severe acute pancreatitis and the risk scores on admission and inflammatory biomarkers in older patients: a retrospective cohort study

**DOI:** 10.1590/1516-3180.2026.3603.14082026

**Published:** 2026-10-23

**Authors:** Nafis Vural, Murat Duyan, Elif Ertas, Hasan Can Guven, Mehmet Akcimen, Osman Gürsoy Unal

**Affiliations:** I MD. Emergency Medicine Specialist, Department of Emergency Medicine, Konya Training and Research Hospital, Baskent University, Konya, Türkiye.; II MD. Associate Professor, Department of Emergency Medicine, Antalya Training and Research Hospital, Antalya, Türkiye.; III PhD. Assistant Professor, Faculty of Medicine, Department of Biostatistics and Medical Informatics, Atlas University, Istanbul, Türkiye.; IV MD. Emergency Medicine Specialist, Department of Emergency Medicine, Antalya Training and Research Hospital, Antalya, Türkiye.; V MD. Associate Professor, Department of Emergency Medicine, Antalya Training and Research Hospital, Antalya, Türkiye.; VI MD. Emergency Medicine Specialist, Department of Emergency Medicine, Antalya Training and Research Hospital, Antalya, Türkiye.

**Keywords:** Pancreatitis, Aged, Emergency Department, Abdominal Pain, Severe Acute Pancreatitis, Morbidity, Diagnosis

## Abstract

**BACKGROUND:**

Acute pancreatitis is an important cause of morbidity and mortality among patients presenting to the emergency department with abdominal pain.

**OBJECTIVES:**

In this study, we aimed to compare the diagnostic performance of the Bedside Index for Severity in Acute Pancreatitis (BISAP) and Emergency Department SpO_2_, Age, and Systemic Inflammatory Response Syndrome (ED-SAS) score for predicting severe acute pancreatitis (SAP) in older patients diagnosed with acute pancreatitis in the emergency department. The predictive performances of inflammatory markers, including the neutrophil-to-lymphocyte ratio, systemic immune-inflammation index (SII), systemic inflammation response index (SIRI), and pan-immune-inflammation value, were also evaluated.

**DESIGN AND SETTING:**

This retrospective cohort study was conducted at a tertiary hospital in Türkiye.

**METHODS:**

Factors associated with SAP were evaluated using univariate and multivariate binary logistic regression analyses; odds ratios with 95% confidence intervals were calculated.

**RESULTS:**

The study included 400 older patients with acute pancreatitis (SAP group, n = 80; non-SAP group, n = 320). The neutrophil-to-lymphocyte ratio, SII, SIRI, pan-immune-inflammation value, ED-SAS score, and BISAP showed good discrimination for SAP, yielding area under the receiver operating characteristic curve values of 0.82, 0.83, 0.85, 0.86, 0.81, and 0.82, respectively. In the multivariate analysis, an SII of > 5796.25 (odds ratio, 13.59), SIRI of > 11.79 (odds ratio, 7.57), ED-SAS score of > 1 (odds ratio, 5.41), and BISAP of > 1 (odds ratio, 2.91) remained independently associated with SAP.

**CONCLUSION:**

The SII, SIRI, ED-SAS score, and BISAP may support early risk stratification for SAP in geriatric pancreatitis. These parameters should be interpreted alongside clinical assessment rather than used as stand-alone diagnostic criteria.

## INTRODUCTION

Acute pancreatitis (AP) is an important cause of morbidity and mortality among patients presenting to the emergency department (ED) with abdominal pain.^1^ AP is also one of the most common gastrointestinal disorders requiring hospitalization.^2^

The most common etiologies of AP include gallstones, hypertriglyceridemia, and excessive alcohol consumption. Other associated factors include certain medications, smoking, iatrogenic biliary tract injury, obesity, and type 2 diabetes. Serum amylase and lipase are routinely used in the diagnostic evaluation of AP. Urinary trypsinogen-2 has a high negative predictive value, whereas serum immunoreactive trypsin and chymotrypsin have also been investigated as potential diagnostic biomarkers.^3^

According to the Revised Atlanta Classification, AP is categorized as mild, moderately severe, or severe, and these categories are closely associated with prognosis.^4^ Although most episodes are mild, mortality in severe acute pancreatitis (SAP) may approach 50%.^4^ Early prediction of SAP is particularly important in older patients, in whom AP may have a more unfavorable clinical course.^5^ ^,^ ^6^ Accordingly, clinical guidelines recommend the early identification of patients at high risk of developing SAP.^7^ ^,^ ^8^

Widely used scoring systems, including the Ranson criteria and the Acute Physiology and Chronic Health Evaluation II score, may be complex and are often completed after hospital admission.^2^ Therefore, simple tools that can be rapidly applied in the ED are needed. In previous studies, the use of the Bedside Index for Severity in Acute Pancreatitis (BISAP),^2^ Emergency Department SpO_2_, Age, and Systemic Inflammatory Response Syndrome (ED-SAS) score, and inflammatory indices such as the neutrophil-to-lymphocyte ratio (NLR), systemic immune-inflammation index (SII), systemic inflammation response index (SIRI), pan-immune-inflammation value (PIV), and the red cell distribution width to total serum calcium ratio have been assessed for predicting SAP early.^2^ ^,^ ^9^ ^,^ ^10^ ^,^ ^11^ ^,^ ^12^ ^,^ ^13^ ^,^ ^14^ ^,^ ^15^ Although the BISAP has been studied in geriatric populations, evidence regarding the use of the ED-SAS score and the inflammatory indices NLR, SII, SIRI, and PIV in this population remains limited.

## OBJECTIVE

The aim of this study was to compare the diagnostic performance of the BISAP, ED-SAS score, NLR, SII, SIRI, and PIV for predicting SAP in older patients with AP who present to the ED.

## METHODS

### Study design and settings

This retrospective cohort study included patients aged 65 or older who presented to the ED with abdominal pain and were diagnosed with AP. Patients admitted between April 1, 2019, and April 1, 2024, were eligible for inclusion.

### Sample size

In this retrospective cohort study, we compared patients with SAP and those with non-severe AP (non-SAP; mild and moderately severe AP). An odds ratio (OR) of 1.5 was considered the minimum clinically meaningful effect size for factors associated with SAP. Based on this assumption, the required sample size was calculated to be 400 patients.^16^ Patients with SAP were matched to patients with mild or moderately severe AP at a ratio of 1 to 4.

### Study protocol

After obtaining approval from the ethics committee, patient records were retrospectively reviewed using the hospital information system. Patients aged 65 or older who presented to the ED of a tertiary training and research hospital between April 1, 2019, and April 1, 2024, were diagnosed with AP when at least two of the following criteria were met: characteristic abdominal pain, a serum lipase concentration of more than three times the upper limit of normal, and imaging findings compatible with AP. The ED-SAS score, BISAP, NLR, SII, and SIRI values; PIV; and other relevant clinical and laboratory data were obtained from the hospital information system. The diagnostic performances of these parameters for predicting SAP were compared.

Patients younger than 65 and those with chronic pancreatitis, pancreatic or hematologic malignancy, traumatic pancreatitis, readmission within 1 week, or incomplete data were excluded.

Cases were classified as mild, moderately severe, or severe according to the Revised Atlanta Classification.^4^ Mild AP was defined by the absence of organ failure and local or systemic complications. Moderately severe AP was defined by transient organ failure resolving within 48 hours and/or local or systemic complications without persistent organ failure. SAP was defined as persistent organ failure lasting more than 48 hours.^4^

### Laboratory analyses

Blood samples were obtained from all patients within 30 minutes of admission to the ED. Complete blood count parameters were measured using an automated hematology analyzer (Coulter Gen-S Hematology Analyzer; Beckman Coulter, Inc., Brea, California). The total leukocyte count, differential leukocyte count, hemoglobin level, hematocrit, platelet count, NLR, SII, SIRI, and PIV were recorded.

The NLR, SII, SIRI, and PIV were calculated as neutrophil count/lymphocyte count, (platelet count × neutrophil count)/lymphocyte count, (neutrophil count × monocyte count)/lymphocyte count, and (neutrophil count × platelet count × monocyte count)/lymphocyte count, respectively.

The BISAP is used for early risk stratification in AP and includes five criteria: a blood urea nitrogen level of > 25 mg/dL, impaired mental status, pleural effusion, age > 60, and the presence of systemic inflammatory response syndrome (SIRS). Each criterion contributes 1 point. Scores of 0 to 2 are associated with a low risk of mortality (< 2%); scores of 3 to 5 indicate a higher risk of mortality (> 15%).

The ED-SAS score incorporates oxygen saturation (SpO_2_), age, and SIRS. The presence of at least two SIRS criteria, an SpO_2_ of < 96%, and age > 60 each contribute 1 point, yielding a maximum score of 3. Thirty-day mortality rates of 4.4%, 12%, and 41.7% for scores of 1, 2, and 3 points, respectively, have been reported.^17^

### Primary purpose

The primary objective was to assess the diagnostic performance of the BISAP, ED-SAS score, NLR, SII, SIRI, and PIV for predicting SAP in older patients presenting to the ED.

### Statistical methodology

Parametric tests were used for continuous measurements due to the suitability of the central limit theorem.^18^ Continuous variables are summarized as mean ± standard deviation and minimum-maximum values, and categorical variables are presented as number and percentage. Student’s t-test was used to compare continuous variables between the SAP and non-SAP groups, and the chi-square test was used for categorical variables.

Receiver operating characteristic curve analysis was performed to determine cutoff values for the NLR, SII, SIRI, PIV, ED-SAS score, and BISAP and to evaluate their diagnostic performance for SAP. Sensitivity, specificity, positive predictive value, negative predictive value, and likelihood ratios were calculated, and areas under the receiver operating characteristics curve (AUCs) with 95% confidence intervals (CIs) are reported. The AUC values were interpreted as follows: 0.9 to 1, excellent; 0.8 to 0.89, good; 0.7 to 0.79, fair; 0.6 to 0.69, poor; and 0.5 to 0.59, failed discrimination. Variables associated with SAP were first evaluated using univariate binary logistic regression; subsequently, they were entered into a multivariate binary logistic regression model. ORs with 95% CIs are reported.

A two-sided p-value of < 0.05 was considered statistically significant. Statistical analyses were performed using MDCalc version 23.0.6 (MDCalc Ltd., Inc., New York) and IBM SPSS Statistics for Windows, version 25.0 (IBM Corp., Armonk, New York).

### Ethical considerations

The study was approved by the ethics committee of the Antalya Training and Research Hospital (decision no. 4/20, dated April 4, 2024), and the requirement for obtaining informed consent was waived because of the retrospective design. The study was conducted in accordance with the Declaration of Helsinki.

## RESULTS

A total of 400 patients with AP were included (SAP group, n = 80; non-SAP group, n = 320). The mean age was 77.3 ± 7.3 years, and the median age was 76 years. Female patients comprised 58.3% of the study population. Of the patients, 4.8% had an impaired mental status, 9.5% had pleural effusion, and 20% had SIRS. Overall, 98.3% of patients were hospitalized and 6% died. The patient and disease characteristics according to AP severity are presented in Table 1.

**Table 1.** Demographic, clinical, and laboratory characteristics of participants according to severity of acute pancreatitis

Parameter	Total N = 400	Non-SAP group n = 320	SAP group n = 80	p value
Age (years), mean ± SD	77.3 ± 7.3	77.1 ± 7.1	78.4 ± 7.5	0.11^†^
Sex, n (%)				
*Female*	233 (58.3)	191 (59.7)	42 (52.5)	0.24^††^
*Male*	167 (41.8)	129 (40.3)	38 (47.5)	
SpO_2_ (%), mean ± SD	96.1 ± 2.3	96.7 ± 1.6	93.8 ± 4.2	< 0.001^†^
Body temperature (°C), mean ± SD	36.3 ± 0.3	36.3 ± 0.3	36.3 ± 0.2	0.95^†^
Heart rate (beats per minute), mean ± SD	86.3 ± 15.1	84.1 ± 14.1	95.2 ± 13.9	< 0.001^†^
Respiratory rate (breath/minute, mean ± SD	15.1 ± 2.5	14.8 ± 2.2	16.2 ± 3.3	< 0.001^†^
Impaired mental status, n (%)	19 (4.8)	0 (0)	19 (23.8)	< 0.001^††^
Pleural effusion present, n (%)	38 (9.5)	11 (3.4)	27 (33.8)	< 0.001^††^
Hospitalization, n (%)	393 (98.3)	314 (98.1)	79 (98.8)	0.71^††^
Number of inpatient days, mean ± SD	6.7 ± 3.8	5.9 ± 2.5	10.2 ± 4.8	< 0.001^†^
Mortality, n (%)	24 (6)	0 (0)	24 (30)	< 0.001^††^
Glucose level (mg/dL), mean ± SD	151.42 ± 41.38	148.47 ± 40.84	153.11 ± 52.51	0.06^†^
Serum sodium level (mEq/L), mean ± SD	136.37 ± 3.61	136.44 ± 3.48	136.08 ± 4.09	0.48^†^
Serum potassium level (mEq/L), mean ± SD	4.13 ± 0.61	4.09 ± 0.56	4.29 ± 0.71	0.009^†^
Urea level (mg/dL), mean ± SD				
Creatinine level (mg/dL), mean ± SD	1.21 ± 0.75	1.12 ± 0.57	1.61 ± 0.82	< 0.001^†^
AST level (U/L), mean ± SD	238.67 ± 151.79	233.05 ± 162.5	261.18 ± 182.5	0.43^†^
ALT level (U/L), mean ± SD	189.05 ± 97.91	187.19 ± 82.11	196.48 ± 92.55	0.72^†^
Bilirubin level, total (mg/dL), mean ± SD	1.98 ± 0.81	1.96 ± 0.73	2.09 ± 0.95	0.57^†^
Bilirubin level, direct (mg/dL), mean ± SD	1.01 ± 0.52	0.99 ± 0.58	1.08 ± 0.62	0.56^†^
Bilirubin level, indirect (mg/dL), mean ± SD				
CRP level (mg/L), mean ± SD	43.39 ± 26.95	40.68 ± 24.62	54.24 ± 30.32	0.06^†^
INR, mean ± SD				
WBC count (10³ μL), mean ± SD	12.45 ± 5.41	11.69 ± 4.67	15.4 ± 6.88	< 0.001^†^
PLT count (10³ μL), mean ± SD	259.15 ± 71.95	253.79 ± 71.49	280.59 ± 70.22	0.003^†^
NEU count (10³ μL), mean ± SD	10.38 ± 4.81	9.43 ± 4.12	14.21 ± 5.21	< 0.001^†^
LYM count (10³ μL), mean ± SD	1.34 ± 0.69	1.46 ± 0.61	0.84 ± 0.53	< 0.001^†^
MON count (10³ μL), mean ± SD	0.74 ± 0.36	0.71 ± 0.32	1.03 ± 0.41	< 0.001^†^
NLR, mean ± SD	12.64 ± 4.04	8.73 ± 4.31	28.29 ± 6.23	< 0.001^†^
SII, mean ± SD	3399.44 ± 404.86	2210.46 ± 428.16	8155.32 ± 628.46	< 0.001^†^
SIRI, mean ± SD	11.01 ± 6.27	6.36 ± 4.51	19.18 ± 9.41	< 0.001^†^
PIV, mean ± SD	2984.28 ± 284.5	1610.39 ± 233.47	8479.88 ± 322.32	< 0.001^†^
SIRS present, n (%)	80 (20)	44 (13.8)	36 (45)	< 0.001^††^
ED-SAS score, mean ± SD	1.43 ± 0.55	1.29 ± 0.49	2.18 ± 0.62	< 0.001^†^
BISAP, mean ± SD	1.65 ± 0.69	1.4 ± 0.58	2.65 ± 0.83	< 0.001^†^

^†^ Student’s t-test; ^††^ Chi-square test (p < 0.05 was considered statistically significant).

SAP, severe acute pancreatitis; SD, standard deviation; SpO_2_, oxygen saturation; AST, aspartate aminotransferase; ALT, alanine aminotransferase; CRP, C-reactive protein; INR, international normalized ratio; WBC, white blood cell; PLT, platelet; NEU, neutrophil; LYM, lymphocyte; MON, monocyte; NLR, neutrophil-to-lymphocyte ratio; SII, systemic immune-inflammation index; SIRI, systemic inflammation response index; PIV, pan-immune-inflammation value; SIRS, systemic inflammatory response syndrome; ED-SAS, Emergency Department SpO_2_, Age, and Systemic Inflammatory Response Syndrome; BISAP, Bedside Index for Severity in Acute Pancreatitis.

Age and sex did not differ significantly between the SAP and non-SAP groups (p = 0.11 and p = 0.24, respectively). The SpO_2_; heart rate; respiratory rate; length of hospital stay; serum potassium and creatinine levels; white blood cell, platelet, neutrophil, lymphocyte, and monocyte counts as well as the NLR, SII, SIRI, PIV, ED-SAS score, and BISAP differed significantly between the groups (all p < 0.05). The temperature; glucose, serum sodium, aspartate aminotransferase, alanine aminotransferase, total and direct bilirubin, and C-reactive protein levels did not differ significantly between groups (all p > 0.05; Table 1).

The severity of pancreatitis was significantly associated with impaired mental status, pleural effusion, mortality, and the presence of SIRS, but not with hospitalization status (Table 1).

The diagnostic performances of the evaluated biomarkers and scores for predicting SAP are summarized in Table 2. The NLR, SII, SIRI, PIV, ED-SAS score, and BISAP showed good discrimination for SAP, yielding AUC values of 0.82, 0.83, 0.85, 0.86, 0.81, and 0.82, respectively (Figure 1).

**Table 2.** Diagnostic performances of the inflammatory indices and clinical scores for predicting severe acute pancreatitis

Index/score	AUC (95% CI, lower-upper)	Cutoff value	Sensitivity %	Specificity %	p value	PPV %	NPV %
NLR	0.82 (0.76-0.88)	> 12.15	74	78	< 0.001	65	92
SII	0.83 (0.8-0.87)	> 5796.25	75	97	< 0.001	72	90
SIRI	0.85 (0.81-0.9)	> 11.79	75	87	< 0.001	65	93
PIV	0.86 (0.82-0.89)	> 3142.89	73	90	< 0.001	70	93
ED-SAS score	0.81 (0.78-0.86)	> 1	81	72	< 0.001	83	86
BISAP	0.82 (0.79-0.87)	> 1	85	70	< 0.001	68	95

AUC, area under the receiver operating characteristic curve; CI, confidence interval; PPV, positive predictive value; NPV, negative predictive value; NLR, neutrophil-to-lymphocyte ratio; SII, systemic immune-inflammation index; SIRI, systemic inflammation response index; PIV, pan-immune-inflammation value; ED-SAS, Emergency Department SpO_2_, Age, and Systemic Inflammatory Response Syndrome; BISAP, Bedside Index for Severity in Acute Pancreatitis.

**Figure 1.**
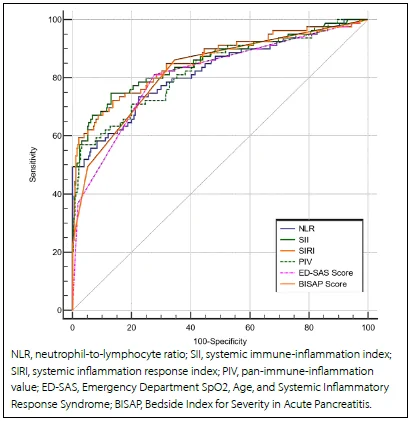
Receiver operating characteristic curves of the inflammatory indices and clinical scores for predicting severe acute pancreatitis.

The univariate associations between the dichotomized predictors and SAP are shown in Table 3 and Figure 2. Patients with an NLR of > 12.15, SII of > 5796.25, SIRI of > 11.79, and PIV of > 3142.89 had 9.68-fold (95% CI: 5.51-16.99), 39.86-fold (95% CI: 18.47-86.01), 18.82-fold (95% CI: 10.35-34.21), and 16.11-fold (95% CI: 8.99-28.87) higher odds, respectively, of having SAP. Similarly, ED-SAS scores of > 1 and BISAPs of > 1 were associated with 11.25-fold (95% CI: 6.10-20.75) and 10.67-fold (95% CI: 5.54-20.55) higher odds, respectively, of having SAP.

**Table 3.** Univariate logistic regression analysis of factors associated with severe acute pancreatitis

Index/score	Odds ratio	95% CI (lower-upper)	p value
NLR (> 12.15)	9.68	5.51-16.99	< 0.001
SII (> 5796.25)	39.86	18.47-86.01	< 0.001
SIRI (> 11.79)	18.82	10.35-34.21	< 0.001
PIV (> 3142.89)	16.11	8.99-28.87	< 0.001
ED-SAS score (> 1)	11.25	6.10-20.75	< 0.001
BISAP (> 1)	10.67	5.54-20.55	< 0.001

Values are presented as odds ratios with 95% CI from univariate binary logistic regression analysis.

CI, confidence interval; NLR, neutrophil-to-lymphocyte ratio; SII, systemic immune-inflammation index; SIRI, systemic inflammation response index; PIV, pan-immune-inflammation value; ED-SAS, Emergency Department SpO_2_, Age, and Systemic Inflammatory Response Syndrome; BISAP, Bedside Index for Severity in Acute Pancreatitis.

**Figure 2.**
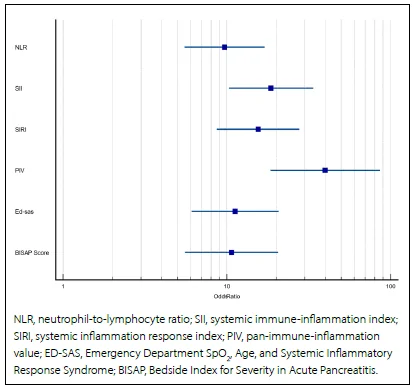
Forest plot of univariate odds ratios with 95% confidence intervals for severe acute pancreatitis.

Variables associated with SAP in the univariate analysis were subsequently evaluated in a multivariate binary logistic regression model. The multivariate model including the NLR, SII, SIRI, PIV, ED-SAS score, and BISAP showed statistical significance (p < 0.001). Using this model, 90% of patients were correctly classified (Table 4).

**Table 4.** Multivariate logistic regression analysis of factors associated with severe acute pancreatitis

Index/score	Odds ratio (95% CI, lower-upper)	p Value (odds)	p Value (model)	Predicted percentage correct
NLR (> 12.15)	1.02 (0.19-1.59)	0.27		
SII (> 5796.25)	13.59 (4.47-41.3)	< 0.001	< 0.0001	0.90
SIRI (> 11.79)	7.57 (1.91-30.07)	0.004		
PIV (> 3142.89)	1.02 (0.15-2.39)	0.47		
ED-SAS score (> 1)	5.41 (2.36-12.41)	< 0.001		
BISAP (> 1)	2.91 (1.27-6.65)	0.01		

Values are presented as adjusted odds ratios with 95% CI from multivariate binary logistic regression analysis.

CI, confidence interval; NLR, neutrophil-to-lymphocyte ratio; SII, systemic immune-inflammation index; SIRI, systemic inflammation response index; PIV, pan-immune-inflammation value; ED-SAS, Emergency Department SpO_2_, Age, and Systemic Inflammatory Response Syndrome; BISAP, Bedside Index for Severity in Acute Pancreatitis.

An NLR of > 12.15 (adjusted OR, 1.02; 95% CI: 0.19-1.59; p = 0.27) and PIV of > 3142.89 (adjusted OR, 1.02; 95% CI: 0.15-2.39; p = 0.47) were not independently associated with SAP in the multivariate analysis, despite their significant univariate associations. An SII of > 5796.25 (adjusted OR: 13.59; 95% CI: 4.47-41.30; p < 0.001), SIRI of > 11.79 (adjusted OR: 7.57; 95% CI: 1.91-30.07; p = 0.004), ED-SAS score of > 1 (adjusted OR: 5.41; 95% CI: 2.36-12.41; p < 0.001), and BISAP of > 1 (adjusted OR: 2.91; 95% CI: 1.27-6.65; p = 0.01) remained independently associated with SAP in the multivariate analysis.

## DISCUSSION

Acute pancreatitis is an important cause of morbidity and mortality in patients presenting to the ED with abdominal pain. Recognizing SAP early is particularly important in older patients. In this study, we evaluated the performance of two clinical scoring systems and four inflammatory indices for predicting SAP in this population.

The NLR is a simple and inexpensive marker of systemic inflammation that reflects neutrophilia and lymphopenia.^19^ Agayev et al.^20^ reported a 21-day survival rate of 96% in patients with an initial NLR of <10.5, compared with 79% in those with higher values. Li et al.^21^ found that an NLR cutoff value of 16.64 predicted mortality with 82.4% sensitivity and 75.6% specificity, and that it remained an independent predictor in the multivariate analysis. Aykan et al.^22^ reported a lower cutoff value of 5.59 for predicting mortality. In the present study, and NLR of > 12.15 showed good discrimination for SAP and was strongly associated with the disease in the univariate analysis; however, it did not remain significant after adjustment for the other biomarkers and scores. Therefore, based on our multivariate findings, the NLR may be useful as an initial screening marker but should not be interpreted as an independent predictor.

The SII reflects the balance between inflammatory activation and immune status. Hökenek^15^ reported that an SII of > 1,243 predicted mortality in AP with 85% sensitivity and 76.4% specificity. In other studies, higher SII values have also been associated with SAP and short-term mortality.^23^ ^,^ ^24^ Yıldız et al.^12^ found that the SII was useful for predicting SAP. In our study, an SII of > 5796.25 showed good discrimination (AUC, 0.83) and remained independently associated with SAP in the multivariate model.

The SIRI, calculated from the neutrophil, monocyte, and lymphocyte counts, reflects systemic inflammatory activity. Bıyık et al.^25^ reported that the SIRI increased progressively with AP severity and discriminated SAP with an AUC of 0.782. Dao et al.^26^ also identified the SIRI as an independent predictor of SAP. In our study, a SIRI of > 11.79 showed good discrimination (AUC, 0.85) and remained independently associated with SAP.

The PIV integrates neutrophil, platelet, monocyte, and lymphocyte counts. Although the PIV has shown prognostic value in other inflammatory and critical illnesses, its role in SAP has not been sufficiently studied.^27^ ^,^ ^28^ In our cohort, a PIV of > 3142.89 showed good discrimination and a strong univariate association with SAP; however, it was not independently associated with SAP in the multivariate analysis. These findings suggest that the PIV may reflect the overall inflammatory burden, but provide limited incremental information when other correlated inflammatory indices and clinical scores are considered simultaneously.

The relatively high cutoff values identified for some inflammatory biomarkers, particularly the SII and PIV, warrant cautious interpretation. These indices combine several complete blood count parameters and may reach markedly elevated values in older patients with severe systemic inflammation, physiological stress, organ dysfunction, or multiple comorbidities. Differences in age distribution, disease severity, timing of blood sampling, laboratory methods, and population characteristics may also explain variation in thresholds across studies. Therefore, the cutoff values identified in this study should not be considered universal limits for clinical decision-making or be used as stand-alone criteria. Rather, they should be regarded as preliminary, cohort-specific thresholds that may complement clinical assessment and established scores such as the ED-SAS score and BISAP. External validation in larger multicenter cohorts is required before routine clinical implementation of these cutoff values.

The ED-SAS score is a practical tool that is based on SpO_2_, age, and SIRS; it was developed for early prediction of mortality and morbidity in AP. Erdoğan et al.^29^ reported that an ED-SAS score of > 1 predicted mortality with 64% sensitivity and 98% specificity (AUC: 0.85). Miller et al.^17^ also demonstrated increasing mortality with higher ED-SAS scores. In our study, an ED-SAS score of > 1 predicted SAP with 81% sensitivity and 72% specificity (AUC, 0.81) and remained independently associated with SAP.

The BISAP enables early assessment of AP severity using clinical parameters available within the first 24 hours of admission. Higher BISAP values have been associated with organ failure, complications, intensive care admission, and mortality.^10^ ^,^ ^30^ ^,^ ^31^ ^,^ ^32^ Satış et al.^33^ reported that a BISAP cutoff value of > 3 predicted SAP in older patients with an 88% sensitivity and 78% specificity (AUC, 0.814). In our study, a BISAP of > 1 showed similar discrimination and remained independently associated with SAP, although the optimal threshold was lower.

Ninety percent of patients were correctly classified using the multivariate model. Nevertheless, this apparent performance should be interpreted with caution because the predictors were partly derived from overlapping hematologic parameters and the model was not externally validated. Among the variables evaluated, the SII, SIRI, ED-SAS score, and BISAP remained independently associated with SAP, whereas the NLR and PIV did not.

To our knowledge, this is the first study to evaluate the use of the SII, SIRI, PIV, and ED-SAS score together for specifically predicting SAP in older patients.

This study had several limitations. First, it was a retrospective, single-center study limited to older patients with AP; this may have restricted the generalizability of the findings. Second, serial or posttreatment biomarker measurements were unavailable. Third, the cutoff values were derived from and evaluated in the same cohort, and no internal or external validation was performed. Finally, the inflammatory indices may have been affected by comorbidities, infection, medications, and other conditions common in older adults. Larger multicenter prospective studies are needed to validate the identified thresholds and assess their clinical utility.

## CONCLUSIONS

Severe acute pancreatitis is associated with substantial mortality in older patients, making early risk stratification essential. The SII, SIRI, ED-SAS score, and BISAP were independently associated with SAP and their use may support early clinical assessment. However, the biomarker cutoff values identified in this study should be interpreted as cohort-specific and require external validation before being used routinely in clinical settings.

## Data Availability

Data supporting the findings of this study are available upon request from the corresponding author, Nafis Vural.
